# Supplementary material for: A mechanism to initiate emergency type 2 myelopoiesis
Source: Nature. 2026 Mar 11;653(8113):212–20. doi: 10.1038/s41586-026-10256-6 (PMC13148993; doi:10.1038/s41586-026-10256-6)
Supplement: Supplementary file 1 — Supplementary Figs 1–4 [file 41586_2026_10256_MOESM1_ESM.pdf]

nature portfolio

https://doi.org/10.1038/s41586-026-10256-6

Supplementary information

A mechanism to initiate emergency type 2 myelopoiesis

In the format provided by the authors and unedited

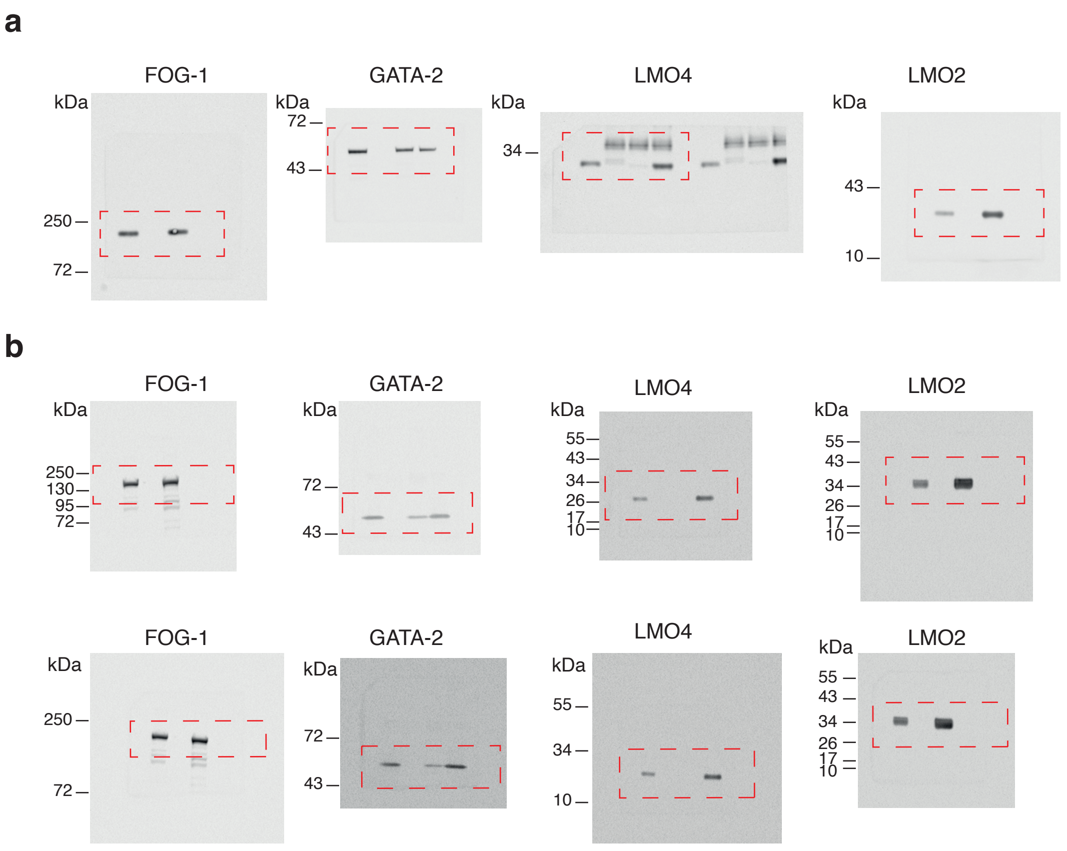

Supplementary Figure 1: Raw Western blot images from **a)** Fig.4b and **b)** Extended Figure.7a. Uncropped blot images are show for each antibody used. Molecular weight markers are indicated. Cropping for final figures is shown as dashed red lines.

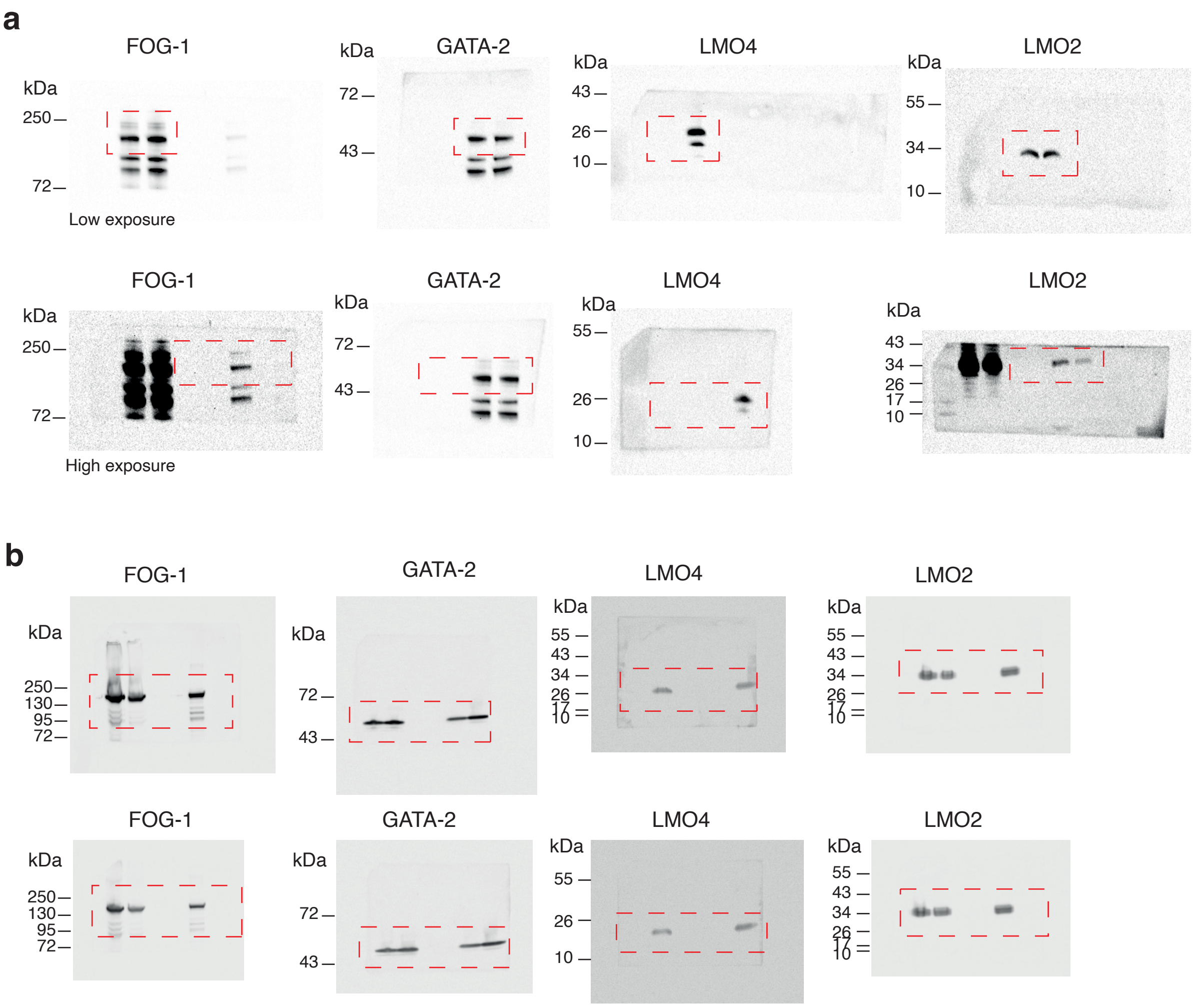

Supplementary Figure 2: Raw Western blot images from **a)** Fig.4c and **b)** Extended Figure.7b. Uncropped blot images are show for each antibody used. Molecular weight markers are indicated. Cropping for final figures is shown as dashed red lines.

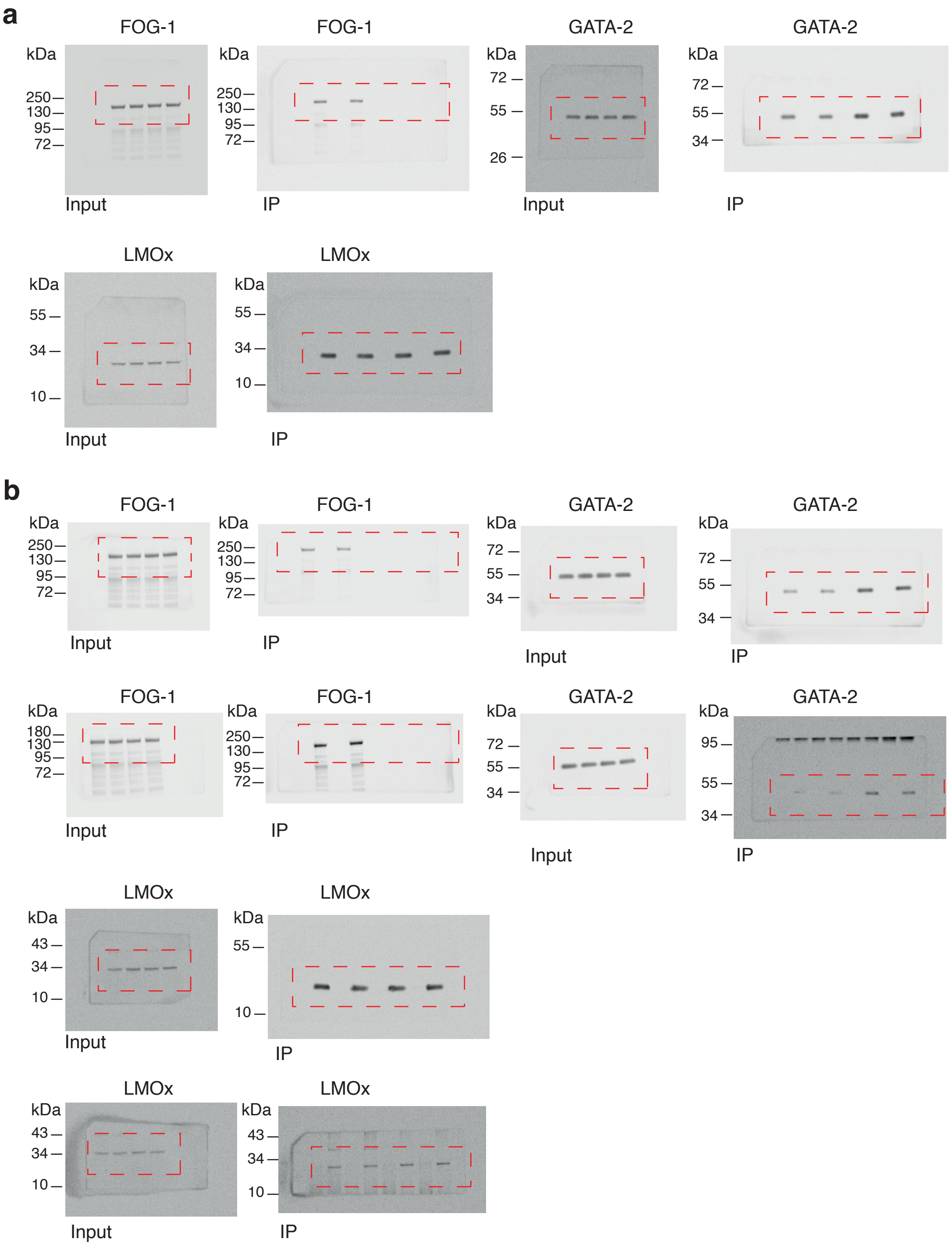

Supplementary Figure 3: Raw Western blot images from **a)** Extended Figure.7f and **b)** Extended Figure.7g. Uncropped blot images are show for each antibody used. Molecular weight markers are indicated. Cropping for final figures is shown as dashed red lines.

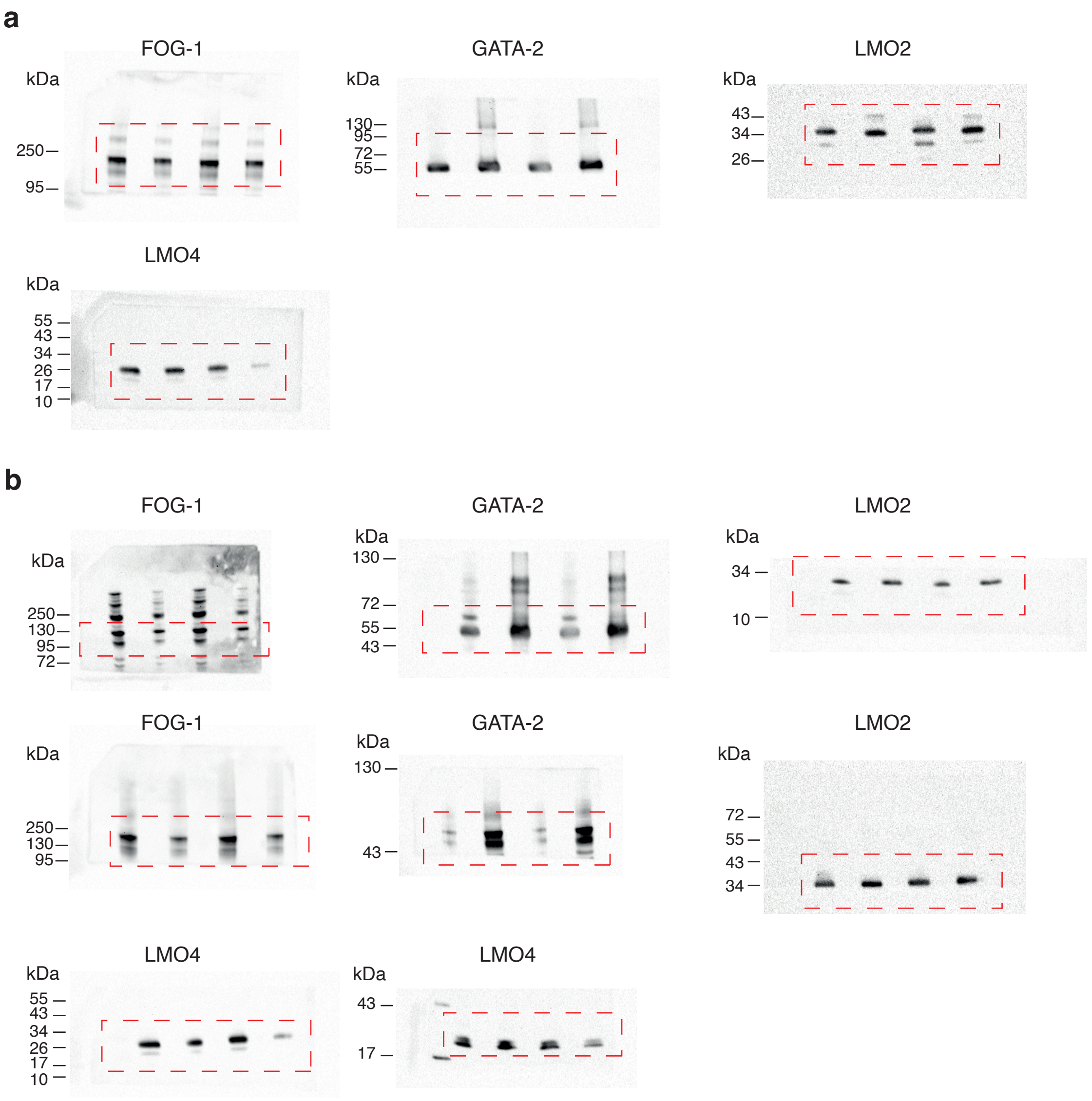

Supplementary Figure 4: Raw Western blot images from **a)** Extended Figure.4f and **b)** Extended Figure.8a. Uncropped blot images are show for each antibody used. Molecular weight markers are indicated. Cropping for final figures is shown as dashed red lines.
