# Supplementary material for: A mechanism to initiate emergency type 2 myelopoiesis
Source: Nature. 2026 Mar 11;653(8113):212–20. doi: 10.1038/s41586-026-10256-6 (PMC13148993; doi:10.1038/s41586-026-10256-6)
Supplement: Supplementary file 2 — Reporting Summary [file 41586_2026_10256_MOESM2_ESM.pdf]

Corresponding author(s): Pr Claus Nerlov
Last updated by author(s): Jan 26, 2026

Reporting Summary

Nature Portfolio wishes to improve the reproducibility of the work that we publish. This form provides structure for consistency and transparency in reporting. For further information on Nature Portfolio policies, see our Editorial Policies and the Editorial Policy Checklist.

Statistics

For all statistical analyses, confirm that the following items are present in the figure legend, table legend, main text, or Methods section.

n/a	Confirmed	
☐	☒	The exact sample size (*n*) for each experimental group/condition, given as a discrete number and unit of measurement
☐	☒	A statement on whether measurements were taken from distinct samples or whether the same sample was measured repeatedly
☐	☒	The statistical test(s) used AND whether they are one- or two-sided<br>*Only common tests should be described solely by name; describe more complex techniques in the Methods section.*
☐	☒	A description of all covariates tested
☐	☒	A description of any assumptions or corrections, such as tests of normality and adjustment for multiple comparisons
☐	☒	A full description of the statistical parameters including central tendency (e.g. means) or other basic estimates (e.g. regression coefficient) AND variation (e.g. standard deviation) or associated estimates of uncertainty (e.g. confidence intervals)
☐	☒	For null hypothesis testing, the test statistic (e.g. *F*, *t*, *r*) with confidence intervals, effect sizes, degrees of freedom and *P* value noted<br>*Give P values as exact values whenever suitable.*
☒	☐	For Bayesian analysis, information on the choice of priors and Markov chain Monte Carlo settings
☒	☐	For hierarchical and complex designs, identification of the appropriate level for tests and full reporting of outcomes
☒	☐	Estimates of effect sizes (e.g. Cohen's *d*, Pearson's *r*), indicating how they were calculated

Our web collection on statistics for biologists contains articles on many of the points above.

Software and code

Policy information about availability of computer code

Data collection	FACS data were collected on BD FACSAria III, BD FACSAria Fusion or BD FACS Fortessa X-20 using FACS DIVA software (v9.0)
Data analysis	FACS data was analysed using Flowio software (version 10.8.1) and subsequent statistical analyses performed using R (v4.2.2)<br>RNA-seq, ATAC-seq and ChIP-seq were analysed as described in the method section, using the following open source software and R packages: Trim Galore ! (version 0.6.5), Salmon (version 1.2.0), DESeq2(version 1.42.0), java GSEAversion 4.2.3), FGSEA(version 1.34.2 http://bioconductor.org/packages/fgsea/), Bowtie2 (version 2.4.1), SAMtools (version 1.17), MACS2 (version 2.1.2), bamCoverage (deepTools 3.5.3), featuresCounts (version 2.0.1) and TOBIAS (version 0.12.1). Single-cell gene expression analysis was performed as described in the method section, using Seurat v5 and ssGSEA (https://rpubs.com/pranali018/SSGSEA). Structural modelling was performed using AlphaFold3 (2024) and visualized using PyMOL Molecular Graphics System (version 2.1.0).

For manuscripts utilizing custom algorithms or software that are central to the research but not yet described in published literature, software must be made available to editors and reviewers. We strongly encourage code deposition in a community repository (e.g. GitHub). See the Nature Portfolio guidelines for submitting code & software for further information.

Data

Policy information about availability of data

All manuscripts must include a data availability statement. This statement should provide the following information, where applicable:

- Accession codes, unique identifiers, or web links for publicly available datasets
- A description of any restrictions on data availability
- For clinical datasets or third party data, please ensure that the statement adheres to our policy

Raw data and proceed data files generated by sequencing are available at Gene Expression Omnibus (GEO) as part of the super-series GSE276830. mm10 (GRCm38) and hg19 (GRCh37) reference genome sequences can be obtained from Gencode (https://www.gencodegenes.org). No homemade code has been used in this study and could be provided upon reasonable request.

Research involving human participants, their data, or biological material

Policy information about studies with human participants or human data. See also policy information about sex, gender (identity/presentation), and sexual orientation and race, ethnicity and racism.

Reporting on sex and gender	*Use the terms sex (biological attribute) and gender (shaped by social and cultural circumstances) carefully in order to avoid confusing both terms. Indicate if findings apply to only one sex or gender; describe whether sex and gender were considered in study design; whether sex and/or gender was determined based on self-reporting or assigned and methods used. Provide in the source data disaggregated sex and gender data, where this information has been collected, and if consent has been obtained for sharing of individual-level data; provide overall numbers in this Reporting Summary. Please state if this information has not been collected. Report sex- and gender-based analyses where performed, justify reasons for lack of sex- and gender-based analysis.*
Reporting on race, ethnicity, or other socially relevant groupings	*Please specify the socially constructed or socially relevant categorization variable(s) used in your manuscript and explain why they were used. Please note that such variables should not be used as proxies for other socially constructed/relevant variables (for example, race or ethnicity should not be used as a proxy for socioeconomic status). Provide clear definitions of the relevant terms used, how they were provided (by the participants/respondents, the researchers, or third parties), and the method(s) used to classify people into the different categories (e.g. self-report, census or administrative data, social media data, etc.) Please provide details about how you controlled for confounding variables in your analyses.*
Population characteristics	*Describe the covariate-relevant population characteristics of the human research participants (e.g. age, genotypic information, past and current diagnosis and treatment categories). If you filled out the behavioural & social sciences study design questions and have nothing to add here, write "See above."*
Recruitment	*Describe how participants were recruited. Outline any potential self-selection bias or other biases that may be present and how these are likely to impact results.*
Ethics oversight	*Identify the organization(s) that approved the study protocol.*

Note that full information on the approval of the study protocol must also be provided in the manuscript.

Field-specific reporting

Please select the one below that is the best fit for your research. If you are not sure, read the appropriate sections before making your selection.

☒ Life sciences ☐ Behavioural & social sciences ☐ Ecological, evolutionary & environmental sciences

For a reference copy of the document with all sections, see nature.com/documents/nr-reporting-summary-flat.pdf

Life sciences study design

All studies must disclose on these points even when the disclosure is negative.

Sample size	Sample sizes were chosen based on the variance previously observed in similar experiments, to allow detection of a 50% difference at P=0.05 with 80% power. Block designs were used to allow variance estimates to be adjusted if necessary
Data exclusions	No data was excluded in this study
Replication	The number of replicate for each experiment is indicated in the figure legend of the manuscript. For RNA-seq, each biological replicate was isolated from individual animal. For ATAC-seq, each biological replicate was generated from a pool of 3 animal. For each experiment, high-throughput sequencing libraries were sequenced in a single library pool to reduce batch effect. For in vitro and in vivo assays, experiment were performed at least twice independently.
Randomization	For in vivo experiment, sex and age-matched animal mice were randomly selected and allocated to experimental groups (i.e: PBS vs IL-33). For other experiment, randomization was not relevant since experimental groups were assigned based on mouse genotype or previous

	treatment.
Blinding	For in vivo experiment, data collection was performed blinded and experimental groups allocated to respective samples at the end of the analysis. For in vitro assays, experimental groups were required to be known during data collection for correct repartition. No analyses required manual counting or subjective analysis were performed and thus not subject to investigator biais.

Reporting for specific materials, systems and methods

We require information from authors about some types of materials, experimental systems and methods used in many studies. Here, indicate whether each material, system or method listed is relevant to your study. If you are not sure if a list item applies to your research, read the appropriate section before selecting a response.

Materials & experimental systems

n/a	Involved in the study	
☐	☒	Antibodies
☐	☒	Eukaryotic cell lines
☒	☐	Palaeontology and archaeology
☐	☒	Animals and other organisms
☒	☐	Clinical data
☒	☐	Dual use research of concern
☒	☐	Plants

Methods

n/a	Involved in the study	
☐	☒	ChIP-seq
☐	☒	Flow cytometry
☒	☐	MRI-based neuroimaging

Antibodies

Antibodies used

SiglecF E50-2440 PE 552126 BD Biosciences
CD150 TC15-12F12.2 APC 115910 Biolegend
FceR1a mars-01 APC 17-5898-82 eBioscience
c-kit 2B8 APC-eFluor780 47-1171-82 eBioscience
CD4 RM4-5 APC-eFluor780 47-0042-82 eBioscience
CD8a 53-6.7 APC-eFluor780 47-0081-82 eBioscience
CD11b M1/70 Alexa Fluor 700 56-0112-80 eBioscience
CD16/32 93 PE-Cy7 25-0161-82 eBioscience
CD19 1D3 PE-Cy7 25-0193-81 eBioscience
CD41 MWRAG30 BV421 133911 Biolegend
SiglecF E50-2440 BV421 562681 BD Biosciences
CD105 MJ7/18 BV421 562760 BD Biosciences
Ter119 TER-119 BV650 116235 Biolegend
CD41 MWReg30 BV650 740504 BD Biosciences
CD105 MJ7/18 BV650 740609 BD Biosciences
Sca-1 D7 BV786 563991 BD Biosciences
Ly-6G/Ly-6C RB6-8C5 Pacific Orange RM3030 Invitrogen
Lmo4 EPR6731(2) NA ab131030 Abcam
Streptavidin NA PE 12-4317-87 eBioscience
CD45.1 A20 FITC 11-0453-85 eBioscience
CD45.2 104 AF700 56-0454-82 eBioscience
CD14 63D3 APC 367118 Biolegend
CD15 W6D3 FITC 323004 Biolegend
CD117 104D2 APC-Fire750 313240 Biolegend
CCR3 5.00E+08 BV421 310714 Biolegend
Fcer1a AER-37 PE-Dazzle 334634 Biolegend
Siglec8 7C9 PE 347104 Biolegend
CD235a HI264 PE-Cy7 349112 Biolegend
CD41 HIP8 BV785 303744 Biolegend
CD34 581 APC 343510 Biolegend
CD38 HIT2 BV421 303526 Biolegend
CD123 6H6 PE-Cy7 306010 Biolegend
CD45RA HI100 BV650 304136 Biolegend
CD131 1C1 PE 306104 Biolegend
anti-GATA2 AF2046 N/A AF2046 R&D
anti-Flag M2 N/A F3165 Sigma
anti-Flag 2368 N/A 2368S Cell Signaling
anti-Myc 71D10 N/A 2278 Cell Signaling
anti-Myc 9.00E+10 N/A MA1-980 Invitrogen
anti-HA 2-2.2.14 N/A 26183 Invitrogen
anti-V5 A190-120A N/A A190-120A Bethyl Laboratories

Validation

Individual antibodies have been titrated to identify the optimal concentration to be used, as previously described (Drissen et al., 2016, Meng et al., 2023)

Eukaryotic cell lines

Policy information about cell lines and Sex and Gender in Research

Cell line source(s)	HEK-293T cell line (Sigma, 12022001-DNA-5UG)<br>HPC-7 cell line (Pinto do O et al., 1998 ; Wilson K.N et al., 2010) was a kind gift from Pr. de Bruijn MFTR.
Authentication	HEK-193T was purchased from the supplier (Sigma) as an authenticated cell line (by STR profiling). HPC-7 cell line was authenticated by differentiation into erythroid, megakaryocyte and myeloid lineages (18 July 2024), as previously described.
Mycoplasma contamination	Cell lines were tested negative for mycoplasm contamination.
Commonly misidentified lines<br>(See ICLAC register)	No commonly misidentified cells were used

Animals and other research organisms

Policy information about studies involving animals; ARRIVE guidelines recommended for reporting animal research, and Sex and Gender in Research

Laboratory animals	8-16 week old mice were used in this study. Mouse strain used are listed bellow:<br>- C57BL/6 CD45.1 (Jackson 002014)<br>- Gata1-EGFP (BAC transgenic; Drissen et al. 2016).<br>- ST2ko (Jackson 039982; Townsend et al., 2000).<br>- NOD.Cg-KitW-41J Tyr+ Prkdcscid Il2rgtm1Wjl/ThomJ (NSGW41; McIntosh B.E., et al, 2015).<br><br>Mice were housed on a 12h light-dark cycle in a standard micro-isolator cages with 45-65% environmental humidity and 19-23°C temperature. Enrichment consisting of red translucent plastic house and crinkle cut naturalistic paper strands.
Wild animals	No wild animals were used
Reporting on sex	Bone marrow transplantation was performed using female recipient mice.
Field-collected samples	This study did not include field-collected samples
Ethics oversight	All animal studies were performed in accordance with the UK Home Office regulations with approval by the University of Oxford Animal Welfare and Ethical Review Body (project license number 30/3359, PP2240412 and PP3246892) and the Francis Crick Institute Animal Welfare and Ethical Review Body (project license number PP8468807), as well as the Irish Health Products Regulatory Authority regulations Licence AE19136/P108 and P196, approved by Trinity College Dublin's Animal Research Ethics Committee

Note that full information on the approval of the study protocol must also be provided in the manuscript.

Plants

Seed stocks	*Report on the source of all seed stocks or other plant material used. If applicable, state the seed stock centre and catalogue number. If plant specimens were collected from the field, describe the collection location, date and sampling procedures.*
Novel plant genotypes	*Describe the methods by which all novel plant genotypes were produced. This includes those generated by transgenic approaches, gene editing, chemical/radiation-based mutagenesis and hybridization. For transgenic lines, describe the transformation method, the number of independent lines analyzed and the generation upon which experiments were performed. For gene-edited lines, describe the editor used, the endogenous sequence targeted for editing, the targeting guide RNA sequence (if applicable) and how the editor was applied.*
Authentication	*Describe any authentication procedures for each seed stock used or novel genotype generated. Describe any experiments used to assess the effect of a mutation and, where applicable, how potential secondary effects (e.g. second site T-DNA insertions, mosiacism, off-target gene editing) were examined.*

ChIP-seq

Data deposition

☒ Confirm that both raw and final processed data have been deposited in a public database such as GEO.

☒ Confirm that you have deposited or provided access to graph files (e.g. BED files) for the called peaks.

Data access links<br>*May remain private before publication.*	Raw data and proceed data files generated by sequencing are available at Gene Expression Omnibus (GEO) as part of the super-series GSE276830
Files in database submission	Both FASTQ and BED files with raw count are provided

Genome browser session
(e.g. UCSC)

n/a

Methodology

Replicates	2 (HEK-293T dataset) or 3 (HPC-7 dataset) biological replicates have been performed per condition.
Sequencing depth	Sequencing depth was 20-40M reads per samples. HEK-293T samples were paired-end and HPC-7 samples were single-end.
Antibodies	Antibodies, including supplier reference number, are listed in supplemental Table 12.
Peak calling parameters	Peak calling was performed using MACS2 (version 2.1.2) with the following parameters: -q 0.05 --nomodel --extsize 200.
Data quality	QC was performed using Trim Galore! version 0.6.5, using QC output file
Software	ChIP-seq was analysed as described in the method section, using the following open source software and Rpackages: Trim Galore ! (version 0.6.5), DESeq2(version 1.42.0), Java GSEA version 4.2.3), FGSEA(http://bioconductor.org/packages/fgsea/), Bowtie2 (version 2.4.1), SAMtools (version 1.17), MACS2 (version 2.1.2), bamCoverage (deepTools 3.5.3) and featuresCounts (version 2.0.1)

Flow Cytometry

Plots

Confirm that:

☒ The axis labels state the marker and fluorochrome used (e.g. CD4-FITC).

☒ The axis scales are clearly visible. Include numbers along axes only for bottom left plot of group (a 'group' is an analysis of identical markers).

☒ All plots are contour plots with outliers or pseudocolor plots.

☒ A numerical value for number of cells or percentage (with statistics) is provided.

Methodology

Sample preparation	Bone marrow and peripheral blood cells were prepared as described in the method section
Instrument	Stained cells were analysed on LSR Fortessa or LSR X-20 flow cytometers (BD Biosciences). Cell sorting was carried-out on FACSAria III, FACSAria Fusion or FACSymphony S6 (BD Biosciences).
Software	Data was collected using FACS DIVA software (v9.0) and analysed using FlowJo software (version 10.8.1) and R (v.4.2.2).
Cell population abundance	Post-sort purity was not feasible for sequencing sample collection as cells were directly sort in lysis buffer.<br>Purity was ensure pre-sort by test-sorting of a relevant cell population.
Gating strategy	FSC-A/SSC-A was used for gating nucleated cells. FSC-A/FSC-H was used for gating singlet cells. Further specific gating strategy is shown in Extended Data Figures of the manuscript.

☒ Tick this box to confirm that a figure exemplifying the gating strategy is provided in the Supplementary Information.
