# Supplementary material for: A mechanism to initiate emergency type 2 myelopoiesis
Source: Nature. 2026 Mar 11;653(8113):212–20. doi: 10.1038/s41586-026-10256-6 (PMC13148993; doi:10.1038/s41586-026-10256-6)
Supplement: Supplementary file 3 — This zipped file contains Supplementary Tables 1–17 and a guide to the tables. [file 41586_2026_10256_MOESM3_ESM.zip › Guide to Supplementary Tables.pdf]

Supplementary Table 1.

Differential gene expression between EMPP isolated from naïve and day 4 post-H.Polygyrus infected mice. Analysis performed using DESeq2; gene name, log2(fold change), p-value and adjusted p-value are indicated for each gene; n=4/condition.

Supplementary Table 2.

Differential gene expression between EMPP isolated from naïve mice or day 11 post-infection with H.Polygyrus. Analysis performed using DESeq2; gene name, log2(fold change), p-value and adjusted p-value are indicated for each gene; n=4/condition.

Supplementary Table 3.

Differential gene expression between BEMP and preMegE expressing empty backbone (Ctrl) isolated from recipient mice. Analysis performed using DESeq2; gene name, log2(fold change), p-value and adjusted p-value are indicated for each gene; n=3/condition.

Supplementary Table 4.

Differential gene expression between EMPP isolated from mice treated with PBS or IL-33 for 2days. Analysis performed using DESeq2; gene name, log2(fold change), p-value and adjusted p-value are indicated for each gene; n=3/condition.

Supplementary Table 5.

Differential gene expression between EMPP over-expressing Lmo4 or empty backbone (Ctrl) isolated from recipient mice. Analysis performed using DESeq2; gene name, log2(fold change), p-value and adjusted p-value are indicated for each gene; n=3/condition.

Supplementary Table 6.

Differential TF motif occupancy predicted by TOBIAS in EMPP over-expressing Lmo4 or empty backbone (Ctrl) at the global chromatin level. Analysis performed using TOBIAS, including TF motif name, p-value as well as TOBIAS statistical test outcome; n=3/condition.

Supplementary Table 7.

Differential TF motif occupancy predicted by TOBIAS in EMPP over-expressing *Lmo4* or empty backbone (Ctrl) at specific BEMP chromatin domains. Analysis performed using TOBIAS, including TF motif name, p-value as well as TOBIAS statistical test outcome; n=3/condition.

Supplementary Table 8.

Differential TF motif occupancy predicted by TOBIAS in EMPP over-expressing *Lmo4* or empty backbone (Ctrl) at specific preMegE chromatin domains. Analysis performed using TOBIAS, including TF motif name, p-value as well as TOBIAS statistical test outcome; n=3/condition.

Supplementary Table 9.

Differential gene expression between EMPP isolated from *Gata2*$^{+/+}$ or *Gata2*$^{D/D}$ mice. Analysis performed using DESeq2; gene name, log2(foldchange), p-value and adjusted p-value are indicated for each gene; n=3/condition.

Supplementary Table 10.

Differential TF motif occupancy predicted by TOBIAS in EMPP isolated from *Gata2*$^{+/+}$ or *Gata2*$^{D/D}$ mice at the global chromatin level. Analysis performed using TOBIAS, including TF motif name, p-value as well as TOBIAS statistical test outcome; n=3/condition.

Supplementary Table 11.

Differential TF motif occupancy predicted by TOBIAS in EMPP isolated from *Gata2*$^{+/+}$ or *Gata2*$^{D/D}$ mice at specific BEMP chromatin domains. Analysis performed using TOBIAS, including TF motif name, p-value as well as TOBIAS statistical test outcome; n=3/condition.

Supplementary Table 12.

Differential TF motif occupancy predicted by TOBIAS in EMPP isolated from *Gata2*$^{+/+}$ or *Gata2*$^{D/D}$ mice at specific preMegE chromatin domains. Analysis performed using TOBIAS, including TF motif name, p-value as well as TOBIAS statistical test outcome; n=3/condition.

Supplementary Table 13.

List of antibodies used for flow-cytometry analysis, including antibody name, clone, conjugated fluorochrome, supplier

Supplementary Table 14.

List of Taqman probes used for microfluidics gene-expression profiling, including target name, assay identification number and targeted species (mouse or human).

Supplementary Table 15.

Normalised gene-expression matrix measured by microfluidics gene-expression, related to *Gata2*^{G320D} mutant analysis (Fig.5f,g). Genotype and sorted cell population is also indicated.

Supplementary Table 16.

Normalised gene-expression matrix measured by microfluidics gene-expression, related to ST2KO/IL-33 chimera analysis (Fig.3c,d). Genotype, treatment and sorted cell population are also indicated.

Supplementary Table 17.

Normalised gene-expression matrix measured by microfluidics gene-expression, related to human $CD34^{+}CD38^{+}CD131^{+}$ progenitor analysis (Fig.3f-h). Seurat clustering and treatment are also indicated.
